# Successful Transcatheter Aortic Valve Replacement in an Elderly Patient with Calcified Bicuspid Aortic Valve with Calcified Raphe: A Case Report

**DOI:** 10.7759/cureus.41850

**Published:** 2023-07-13

**Authors:** Siva Sagar Taduru, Madhuri Ramakrishnan, Hema Pamulapati

**Affiliations:** 1 Cardiovascular Medicine, University of Kansas Medical Center, Kansas City, USA; 2 Nephrology, Hays Medical Center, Hays, USA; 3 Nephrology, University of Kansas Medical Center, Kansas City, USA; 4 Cardiovascular Disease, University of Kansas Medical Center, Kansas City, USA

**Keywords:** tavr (transcatheter aortic valve replacement), aortic valve insufficiency, aortic valve replacement(avr), aortic stenosis (as), bicuspid aortic valve disease

## Abstract

At present, transcatheter aortic valve replacement (TAVR) is not only used in high-surgical-risk patients with aortic stenosis (AS), but its use has also been extended to low-risk patients, resulting in its increasing utilization in patients with bicuspid aortic valve (BAV). BAV however presents unique challenges for TAVR due to its distinct valvular anatomy, and surgical aortic valve replacement (SAVR) remains the primary recommended method of aortic valve replacement in patients with BAV. Nonetheless, observational data have been quickly accumulating regarding the successful use of TAVR in BAV. Here, we present a case of a 73-year-old female who presented with heart failure symptoms and was found to have severe AS and BAV with calcified raphe (Sievers 1a). Due to her age and complicated medical history, including coronary artery disease and chronic kidney disease, she was considered to be at intermediate surgical risk (Society of Thoracic Surgeons (STS) score 5.4%) and underwent TAVR with the successful deployment of a 29 mm Edwards SAPIEN valve (Edwards Lifesciences, California, USA). A post-procedure echocardiogram confirmed the appropriate placement of the prosthesis without any valvular or paravalvular regurgitation. This case, therefore, adds to the growing body of evidence regarding the use of TAVR in patients with BAV despite anatomical challenges.

## Introduction

Recent advancements have extended the use of transcatheter aortic valve replacement (TAVR) for treating aortic stenosis (AS) to low-risk patients, as established by randomized controlled trials [1]. However, these trials excluded patients with unicuspid, bicuspid, or non-calcified aortic valves and those with severe aortic regurgitation, among others [1]. Despite these exclusions, about 3-10% of patients undergoing TAVR have a bicuspid aortic valve (BAV), a common congenital heart disease [2,3]. In addition, TAVR has been applied in severe aortic valve regurgitation cases as a compassionate/off-label use, including those with a left ventricular assist device (LVAD) [4].

Although the Food and Drug Administration (FDA) approved TAVR for low-risk patients in 2019, randomized data comparing TAVR and surgical aortic valve replacement (SAVR) in BAV patients is lacking, with available data being predominantly observational. BAV presents unique challenges for TAVR due to distinctive annular dimensions, elliptical orifice geometry, eccentric calcification, high calcium scores, and a high risk of conduction disturbances. These factors can increase complications, such as paravalvular regurgitation, and the rates of permanent pacemaker implantation [5].

Observational studies indicate improved outcomes with TAVR for bicuspid AS over time due to advancements in device technology, imaging modalities, and implantation techniques. Computed tomography (CT) scans, now routinely performed for procedure planning, and a better understanding of BAV anatomies have likely enhanced patient selection [6,7]. Among patients with BAV, those who have valves that are heavily calcified and have a calcified raphe are associated with increased mortality while undergoing TAVR [8]. Here, we present a case of a 73-year-old female with BAV with calcified raphe who underwent successful TAVR.

## Case presentation

**Figure 1:**
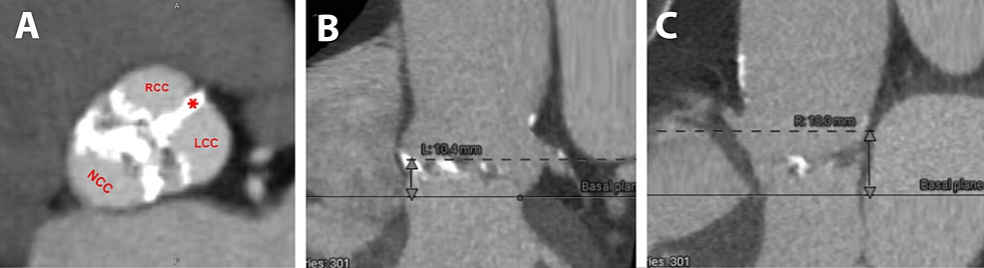
Pre-procedural transcatheter aortic valve replacement computed tomography angiogram measurements (1) A - Short axis of the bicuspid aortic valve; B - left coronary artery height; C - right coronary artery height RCC: right coronary cusp; LCC: left coronary cusp; NCC: non-coronary cusp; *: fused RCC and LCC with calcified raphe

**Figure 2:**
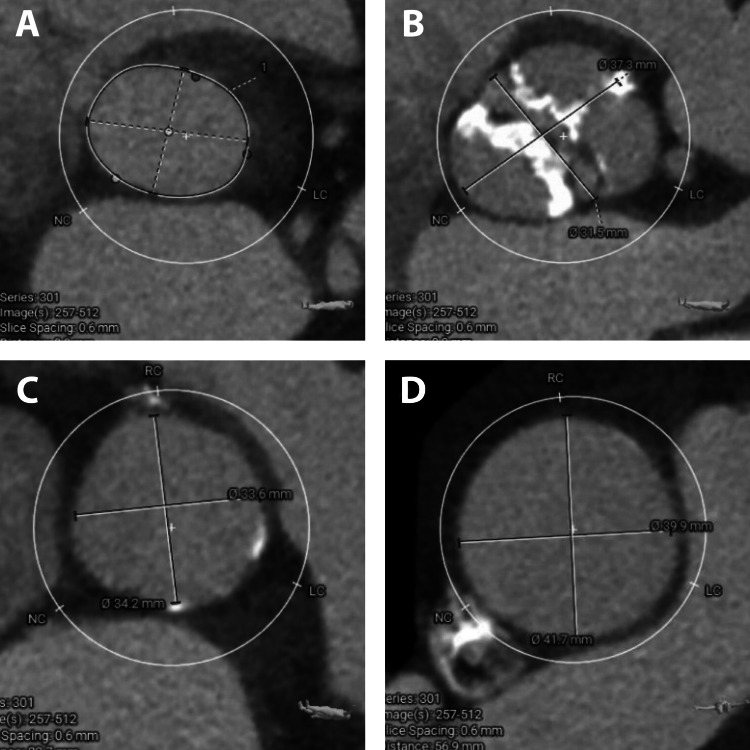
Pre-procedural transcatheter aortic valve replacement computed tomography angiogram measurements (2) A - Aortic annulus measurements, B - sinus of Valsalva measurements, C - sinotubular junction measurements, D - proximal ascending aorta measurements

A 73-year-old female with a complex medical history, including coronary artery disease, BAV (Sievers 1a), chronic kidney disease stage III, hypertension, hyperlipidemia, atrial fibrillation, and prior stroke, was hospitalized due to progressive dyspnea and edema of four-week duration. After treating her decompensated heart failure with intravenous diuretics, she underwent an echocardiogram that revealed severe calcific BAV stenosis with a peak velocity of 4.7 m/s, a mean gradient of 54 mmHg, aortic valve area of 0.77 cm², and a dimensionless index of 0.2 along with mild eccentric aortic valve regurgitation. She had a normal left ventricular size with mild concentric left ventricular hypertrophy, normal left ventricular ejection fraction with an estimated ejection fraction of 60-65%, grade 3 diastolic dysfunction, normal right ventricular size, and systolic function. She underwent pre-procedural TAVR CT angiography (CTA) that confirmed the presence of a calcified BAV with fused right and left coronary cusps, along with a calcified raphe (Figure 1A). Notably, no calcium deposition was observed in the left ventricular outflow tract. Measurements were taken, including the heights of the left coronary artery (10.4 mm), right coronary artery (18.0 mm) (Figure 1B, 1C), and the sinuses of Valsalva (34 x 37 mm), aortic annulus (29 x 32 mm, area: 655.8 mm2, perimeter: 91.8 mm), sinotubular junction (34 x 34 mm), and proximal ascending aorta (40 x 41.7 mm) (Figure 2A, 2D).

CTA analysis was completed using 3mensio Aortic Valve software (PIE Medical Imaging, Netherlands). A multidisciplinary heart valve team evaluated the patient's case, and it was decided to proceed with the TAVR procedure given the patient's age and medical comorbidities putting her at intermediate risk based on the Society of Thoracic Surgeons Predicted Risk of Mortality (STS PROM) score of 5.4% for SAVR.

**Figure 3:**
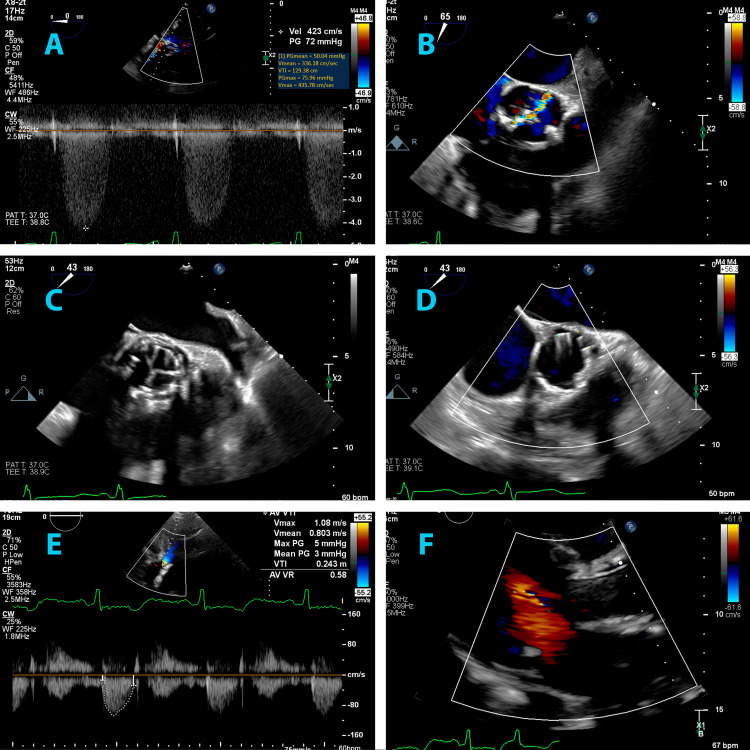
Intraprocedural TEE and post-procedural TTE A - Intraprocedural TEE confirming velocities consistent with severe aortic stenosis, B - intraprocedural TEE showing aortic regurgitation, C - intraprocedural TEE showing TAVR post-deployment, D - intraprocedural TEE showing lack of valvular or paravalvular regurgitation, E - post-procedural TTE showing normalized aortic valve velocities and gradients, F - post-procedural TTE showing lack of valvular or paravalvular regurgitation TEE: transesophageal echocardiogram; TTE: transthoracic echocardiogram; TAVR: transcatheter aortic valve replacement

Intraprocedural transesophageal echocardiography (TEE) verified a peak velocity of 4.36 m/s, accompanied by a mean gradient of 50 mmHg (Figure 3A). The left common femoral vein was accessed to insert a 6 French sheath, facilitating the advancement of a temporary pacer into the right ventricle (RV). Similarly, the left common femoral artery was accessed, where a 6 French sheath was introduced and a 5 French pigtail catheter was prepared for aortograms. Furthermore, the right femoral artery was accessed to advance a 14 French Edwards sheath. After the successful crossing of the aortic valve, pressure measurements were recorded. Before balloon valvuloplasty, a guide was introduced into the left main coronary artery (LMCA), while a Sion wire (Asahi Intecc USA, Inc., USA) was placed as a preventive measure to address concerns regarding potential obstruction at the coronary origin by the native aortic valve leaflet displacement following TAVR deployment, along with a 4 x 8 mm resolute Onyx stent (Medtronic, Ireland). This was performed as our patient had some of the risk factors associated with a higher risk of coronary obstruction, such as being female, having LCMA height <12 mm, and using a balloon-expandable valve. Subsequently, balloon valvuloplasty was conducted using a 23 x 45 mm True balloon (Becton, Dickinson and Company, USA). Following this step, a 29 mm Edwards SAPIEN valve (Edwards Lifesciences, California, USA) was meticulously positioned and deployed under rapid ventricular pacing. A post-deployment TEE confirmed the appropriate placement of the prosthesis without any valvular or paravalvular regurgitation. Post-deployment, the peak velocity across the aortic valve was measured at 0.86 m/s, with a mean gradient of 2 mmHg (Figure 3C, 3D, 3E, 3F). After verifying that there was no obstruction of the LMCA by coronary angiography and Intravascular ultrasound (IVUS), the stent delivery system was then removed without deploying the stent. Finally, ProGlide sutures (Abbott, USA) were deployed after removing the sheath from the right femoral artery. A pelvic angiogram was performed to ensure no arterial injury or leaks were present. The patient was discharged on the third day of hospitalization.

## Discussion

Over the years, indications for TAVR have been extended to include low surgical risk and therefore younger population, resulting in the performance of TAVR in patients with BAV [1]. This has led to an increase in the use of TAVR for patients with BAV, although SAVR is the preferred method due to its high technical success and unique valve anatomy [3]. Our patient was deemed to be at intermediate risk for SAVR. She also had calcified BAV with calcified raphe, which has previously been shown to have relatively worse outcomes in patients undergoing TAVR. Despite this and consistent with previous observational studies that have shown improving outcomes in these types of cases, we elected to perform TAVR in this patient. Thus, our case report of a 73-year-old, an intermediate-surgical-risk patient with high-risk BAV features who underwent successful TAVR, adds to the growing body of evidence regarding the successful utilization of TAVR in BAV after careful consideration.

BAV is the most common congenital heart anomaly, seen in about 1-2% of the population, and BAV cases represent about 50% of all patients evaluated for aortic valve replacement (AVR) [5]. Although SAVR is the preferred method of AVR in patients with BAV, in the TAVR era, approximately 3-10% of patients that undergo TAVR have BAV [2,3].

BAV is known to pose unique challenges due to its anatomical and pathophysiological characteristics, such as large annuli, elliptical orifice geometry, increased cusp calcification often with a bulky and asymmetric calcification, presence of calcified raphe, aortic root, and ascending aorta dilation [5,8]. However, observational studies suggest that the continued evolution of device technology, imaging modalities, and procedural techniques have improved TAVR outcomes for patients with BAV [6,7]. This observation is consistent with the outcome of our case report. CT scans, now routinely performed for procedure planning, offer essential insights into BAV anatomies, enhancing patient selection and planning [5]. Similarly, our patient's successful procedure underscores the importance of meticulous pre-procedural planning, which identified a calcified BAV and allowed for the selection of the appropriate size of the TAVR prosthesis.

Although rare, acute coronary occlusion (ACO) during TAVR is a critical event, with a prevalence of under 1% in native aortic valves and 3.5% in valve-in-valve procedures [9,10]. It chiefly impacts the LMCA through native aortic valve leaflet displacement. Predictive anatomical factors encompass coronary height below 12 mm and sinus of Valsalva diameter under 30 mm [10]. Risk factors also include advanced age, female sex, absence of prior coronary bypass surgery, valve-in-valve procedures, and the use of balloon-expandable valves [10]. This can complicate up to 4.6% of TAVR cases in patients with BAV [11]. Previous studies have proposed pre-emptive wiring of the coronary ostia and eventual stent deployment as a potential preventive strategy to prevent ACO [12]. Our patient had some of these risk factors, including advanced age, female sex, LMCA height of 10.4 mm, and the use of a balloon-expandable valve. Therefore, we employed this strategy for LMCA protection.

## Conclusions

Our case report describes a single patient experience and cannot definitively address the overall risk-benefit ratio of TAVR in BAV patients with similar characteristics. Randomized clinical trials are necessary to further elucidate these complex relationships and to optimize procedural and post-procedural management. Despite these limitations, this case highlights the potential of TAVR as a therapeutic option in patients with BAV, even in complex scenarios, such as heavily calcified valves with calcified raphe, offering avenues for a broader range of treatment strategies for these patients in the future.
